# The added value of preoperative thoracic CT imaging in the management of T1a renal cell carcinoma

**DOI:** 10.1177/02841851251411039

**Published:** 2026-01-29

**Authors:** Elin Gullberg Bohlin, Maria Hermann, Tomas Thiel, Per-Olof Lundgren

**Affiliations:** 1 Department of Clinical Science, 27106 Intervention and Technology, Karolinska Institute, Stockholm, Sweden; 2 Department of Surgery, Falun Regional Hospital, Falun, Sweden; 3 Department of Urology, Karolinska University Hospital, Huddinge, Stockholm, Sweden

**Keywords:** Renal cancer, renal cell carcinoma, preoperative routine, computed tomography

## Abstract

**Background:**

In Sweden, approximately 1300 patients are diagnosed with renal cell carcinoma (RCC) every year. The use of a computed tomography (CT) scan of the thoracic cavity in the preoperative work up of kidney cancer has increased in Sweden, and current national guidelines recommend that all patients, regardless of tumor size, should be evaluated this way

**Purpose:**

To investigate the need for the preoperative routine to include a CT scan of the thoracic cavity when investigating renal masses 4 cm or smaller.

**Material and Methods:**

Between 2017 and 2022, 496 patients at a university hospital and a regional hospital received treatment with curative intent for T1a tumors. Patient data and pathological findings were registered from patient records.

**Results:**

Median follow-up was 38 months. A total of 260 patients were examined with a preoperative CT scan of the thoracic cavity without pathology: 46 had not been scanned, 118 had indeterminate lesions, and metastasis was suspected in two cases. During follow-up, six patients had local relapse and none was diagnosed with lung metastasis. In no case did the preoperative CT of the thoracic cavity contribute to an early discovery of lung metastases.

**Conclusion:**

Our conclusion is that a chest CT scan is superfluous in the preoperative work-up. The cost, and the time to treatment, could be reduced by precluding the chest CT in the preoperative work up for small renal tumors.

## Introduction

In Sweden, approximately 1300 patients are diagnosed with renal cell carcinoma (RCC) every year (1). A majority of new cases are discovered incidentally, when investigating other abdominal diseases and symptoms (2). In addition, a large fraction is small (≤4 cm) and up to 30% are found to be benign (1,3). Approximately 24% of patients diagnosed with RCC have T1a disease (4).

The use of a computed tomography (CT) scan of the thoracic cavity in the preoperative work up of kidney cancer has increased in Sweden, and current national guidelines recommend that all patients, regardless of tumor size, should be evaluated this way (1,5). This recommendation contrasts from European guidelines, where the chest CT “may be omitted in patients with cT1a and cN0 without systemic symptoms, e.g. anemia or thrombocythemia, due to the low incidence of lung metastasis (<1%) in this cohort” (6). This is supported by studies showing that the use of a nomogram can identify a patient cohort with low risk of disease and <1% risk of metastasis (7,8). On the other hand, American guidelines recommend that metastatic evaluation should include chest imaging to evaluate for possible thoracic metastases as a clinical principle (9). This is in alignment with data from the National Swedish Kidney Cancer Register (NSKCR), which show a risk of 7.5% for local recurrence or metastasis within 5 years of intervention of T1a tumors (10).

The use of CT examinations is increasing (11). Given the non-negligible effects of radiation on population health, it is important to ensure that these examinations are better justified to minimize population exposure and reduce patient anxiety. Another key consideration is the impact on healthcare costs and the potential displacement effect caused by redundant radiological examinations.

The aim of the present study was to evaluate the clinical necessity of preoperative thoracic staging using CT in patients with small, incidentally detected renal lesions. This study seeks to address a persisting gap in the literature and to provide evidence that may refine current preoperative evaluation guidelines.

## Material and Methods

Ethical approval was obtained from the Swedish National Ethical Review Authority (reference nos. 2022-01981-01 and 2024-02023-02). Only institutional-level data were extracted from the Swedish Kidney Cancer Registry, which is why the study was exempt from a registry-specific data application process. The manuscript was drafted using the Strengthening the Reporting of Observational Studies in Epidemiology (STROBE) statement (12).

Relevant data were collected from the Swedish National Kidney Cancer Registry (with a coverage rate of 96%) and from patient records. Age, sex, preoperative CT scan of the thorax, X-ray findings, date and type of treatment, relapse (location and time), tumor size, and follow-up time (cause of death, where relevant) were recorded.

### Patients

Between 2017 and 2022, a total of 496 patients were treated for T1a renal cancer at two centers, one university hospital and one regional hospital. Patients were treated with either thermal ablation, minimally invasive surgery, or conventional open surgery (either nephrectomy or partial nephrectomy). Of the 496 patients, 435 were treated at the university hospital and 61 at the regional hospital. We included previously treated patients who were aged older than 18 years at the time of the intervention. Clinical practice and national guidelines at this time were to routinely evaluate all patients with a suspected renal mass using CT of the thoracic cavity.

The exclusion criteria were residual tumor after ablation (n = 1), wrong tumor characteristics (n = 41), errors in registration (n = 1), tumors in transplanted kidneys (n = 1), patients with synchronous malignancies (n = 14), or if follow-up data were missing (n = 12). In total, 426 patients could participate in the study.

### Data extraction and radiology assessment

Radiology was re-evaluated preoperatively by experienced urologists and radiologists. The majority of cases were discussed in a multidisciplinary meeting. Preoperative intrathoracic findings were stratified by “no evidence of pathology,” “indeterminate lesions,” and “suspicious lesions,” derived from the original radiology report or by a secondary assessment during a multidisciplinary meeting (Table 2).

### Surgical interventions

All patients were treated according to tumor and patient characteristics with either thermal ablation, laparoscopic surgery, or conventional open surgery (either nephrectomy or partial nephrectomy) (Table 1).

**Table 1.** Baseline characteristics of the study cohort.

Treatment modality	Total (n = 425)	Radical nephrectomy (n = 60)	Partial nephrectomy (n = 178)	Thermal ablation (n = 188)
Follow-up (months)	38 (6–84)	37.5	38	37.5
Age (years)	68 (26–89)	71	61	73
Male (%)	68	67	73	66

Values are given as median (range) unless otherwise indicated.

### Follow-up

Follow-up started at the time of surgery and at least one CT scan of the thorax was performed before the end of the follow-up. Endpoints were time of recurrence, death, or last contact with the urology department (median = 38 months, range = 6–84 months).

## Results

In total, 426 patients (68% men, 32% women; median age at time of treatment = 68 years; age range = 26–89 years) were included from the two centers, one university hospital and one regional hospital. Median follow-up was 38 months (range = 6–84 months).

In total 6 (1.4%) patients were diagnosed with recurrent disease, none in the thoracic cavity. All recurrences were diagnosed using CT. Five were local recurrences in the ipsilateral kidney after nephron-sparing interventions and one recurrence occurred in a regional lymph node.

In total 39 (9.2%) patients died during follow-up, all of them from other causes. Two of the patients who died during follow-up were indeed diagnosed with recurrent disease, one with lymphatic spread and one with local recurrence. Both recurrences were diagnosed radiologically without histological verification (Tables 2–4).

**Table 2.** Initial findings on CT of the thoracic cavity.

Operation	n	No evidence of pathology	Indeterminate lesion	Suspected metastasis	No preoperative CT	Median follow-up (months)	Recurrence
Nephrectomy	60	42	13	0	6	37.5	1^*^
Kidney resection	178	110	55	2	8	38	1^†^
Ablative therapy	188	108	50	0	32	37.5	4*
Total	426	260	118	2	46		6

* Local recurrence.

^†^ Lymph nodes.

**Table 3.** Number of recurrences stratified by preoperative CT scan.

	n	Lung metastasis	Local recurrence	Lymph nodes
Included in study	426	0	5 (1.2)	2 (0.5)
No previous CT	46	0	3 (6.5)	1 (2.17)
No evidence of pathology	260	0	0	1 (0.38)
Suspected metastasis	2	0	1 (50)	0
Indeterminate lesion	118	0	0	0

Values are given as n or n (%) unless otherwise indicated.

**Table 4.** Findings on the preoperative thorax CT in the group with a recurrence.

	n	Suspected metastasis	Indeterminate lesion
Lung metastasis	0	0	0
Local recurrence	5	1	0
Lymph nodes	1	0	0

## Discussion

A CT scan of the thoracic cavity is likely not necessary for the staging of small incidentally found renal lesions. There were no recurrences in the pulmonary viscera and hence there was no association between preoperative findings on CT scans of the thoracic cavity and specific disease recurrence to the thoracic cavity.

Up to 30% of all small renal masses are benign (1,3), which alone makes routinely examining all patients with a chest CT scan preoperatively an over-examination and leads to subjecting these patients to unnecessary radiation by obtaining preoperative CT of the chest.

In addition, it is estimated that 1.2% of cancers annually are induced by radiological scans (11). Data from the USA suggest that the number of cancers that are radiologically induced might be as high as 2% (11). It is also known that the risk for radiation-associated cancer increase with lower age and affects women to a higher degree (13). The low detection rate of pulmonary metastasis combined with the obvious disadvantages of unnecessary scans prompts a discussion concerning the rationale for given recommendations in guidelines and urges individualized assessment by the clinician, taking into account other variables than tumor data alone (13,14).

Today EAU guidelines and AUA guidelines (6,9) contradict each other concerning whether or not a scan of the thoracic cavity should be recommended for the staging of small renal masses. Inevitably, the data on which the guidelines are based are older and do not completely reflect the increasing number of CT scans that are performed or new diagnostic tools. This highlights the need to critically reflect on the clinical experience and, as in this study, question the need for additional examinations on a routine basis.

In a clinical setting where resources are limited, it is important to focus on important examinations. Performing unnecessary staging of T1a renal tumors will not only be costly but might also have a displacement effect (15).

Patients with small renal masses experience additional stress during watchful waiting and additional examinations could have an adverse effect on the psychological health of a patient under investigation for a suspected renal tumor (16). Even indeterminate findings on chest CT can have a negative psychological effect on patients (17).

There is a risk that a patient with cT1a disease on the preoperative scan will be upgraded in the pathology report. However, this scenario is rare, and performing a chest CT shortly after surgery should be adequate, as the likelihood of metastasis in this group is low. In case of a pathology report suggesting rare and/or very aggressive disease, individual assessment of every case is prudent. There are cases, however rare, where small lesions also lead to systemic disease (10). It is unknown whether these cases showed suspected lung metastasis on the preoperative CT scan or if evidence of systemic disease could only be found postoperatively. In our database, no case of primary M1 disease was found in the cohort with tumors less than 4 cm in size.

The present study has some limitations. First is the low rate of events, making estimations of hazard unplausible. Nonetheless, our results support earlier evaluations that small tumors rarely metastasize and that staging of M1 disease can be deferred until the first postoperative scan (18). Another limitation is the selection of patients. A majority of the cohort is defined from a tertiary referral center making generalization problematic. Even so, this should most likely yield a more advanced cohort with putatively higher risk of metastasis or recurrence. Previous studies have also noted that tumor size is the strongest factor predicting the risk for metastasis. The tumor-related risk significantly increases above 3 cm, revealing a shortcoming in the TNM classification that labels all tumors under 4 cm as T1a. The association between tumor size and location of metastasis was not evaluated (10,19).

The study's small population and retrospective design constitute notable limitations. Since all patients in this cohort were treated (with either ablative therapy or surgery), there is an inherent risk for selection bias, i.e. this cohort has been considered suitable for active treatment. As a result, these findings should only be generalized to this population. Furthermore, conclusions drawn from this study are limited to the preoperative evaluation of the thoracic cavity. Despite potential risks of missing data and bias, the high estimated completeness of the Swedish Cancer Registry (96%) lends credibility to the findings. Further discussions prompted by the results include possible revisions of recommended follow-up protocols and evaluation of associations between indeterminate lesions and subsequent disease recurrence.

In conclusion, a CT scan of the thoracic cavity may be unnecessary before treatment decisions for small renal masses (<4 cm). Our findings support current guidelines advising against preoperative chest CT in this patient population. Further studies with larger sample sizes are warranted to strengthen the evidence in this area.
